# Functional benefits of corticosteroid and IVIG combination therapy in a coronary artery endothelial cell model of Kawasaki disease

**DOI:** 10.1186/s12969-020-00461-6

**Published:** 2020-10-06

**Authors:** Takashi Inoue, Shokei Murakami, Kenji Matsumoto, Akio Matsuda

**Affiliations:** 1 Department of Allergy and Clinical Immunology, National Research Institute for Child Health and Development, 2-10-1 Okura, Setagaya-ku, Tokyo, 157-8535 Japan; 2 Department of Pediatrics, Jikei University School of Medicine, Tokyo, Japan; 3 Department of Pediatrics, Toyama University School of Medicine, Toyama, Japan

**Keywords:** Kawasaki disease, Coronary artery endothelial cells, Corticosteroid, IVIG, IgG, HMGB1, IL-1α, IL-6, G-CSF

## Abstract

**Background:**

Kawasaki disease (KD) is the most common pediatric systemic vasculitides of unknown etiology. Recent clinical studies led to reappraisal of the usefulness of initial combination therapy of intravenous immunoglobulin (IVIG) plus a corticosteroid for patients with severe KD. However, the molecular mechanisms underlying the clinical benefits of that combination therapy remain unclear. Here, we used cultured human coronary artery endothelial cells (HCAECs), as a mimic of KD, to study the possible mechanisms responsible for the clinical benefits of adding a corticosteroid to standard IVIG therapy for patients with severe KD.

**Methods:**

HCAECs were stimulated with TNF-α, IL-1α or IL-1β in the presence and absence of high-dose IgG and/or dexamethasone (DEX). The mRNA and protein concentrations for high-mobility group box-1 (HMGB1), IL-1α, IL-6 and granulocyte-colony stimulating factor (G-CSF) in the culture supernatants were measured by quantitative PCR (qPCR) and ELISA, respectively. Apoptosis was evaluated by the caspase 3/7 activities.

**Results:**

DEX, but not IgG, significantly inhibited apoptosis caused by inflammatory stimuli, resulting in effective reduction of HMGB1 and IL-1α protein release by HCAECs. As previously reported, DEX or IgG alone significantly suppressed TNF-α-induced production of IL-6 and G-CSF and mRNA expression, but induction of those cytokines by IL-1 s (IL-1α and IL-1β) was resistant to high-dose IgG.

**Conclusions:**

A corticosteroid can effectively inhibit the release of HMGB1 and IL-1α, which may be involved in IVIG resistance in KD. Since high-dose IgG does not have such beneficial anti-cytotoxic effects, adding a corticosteroid to standard IVIG therapy may help prevent the progression of IVIG resistance in KD.

## Background

Kawasaki disease (KD), first described by Dr. Tomisaku Kawasaki in 1967 in Japan [1], is one of the most common pediatric systemic vasculitides of unknown etiology. Since that first report, the morbidity rate of KD in Japan has continued to rise, and currently, there are more than 15,000 new patients annually [2]. The most serious clinical issue in KD is the formation of coronary artery lesions (CALs) due to severe inflammation of the coronary arteries. Intravenous immunoglobulin (IVIG) has been used as the first-line standard treatment for KD and is highly effective in almost 80% of patients. On the other hand, the remaining patients are resistant to initial IVIG treatment, and they are at high risk for CALs compared with IVIG-responsive KD patients [3]. The most common cause of acquired heart disease in childhood in developed countries is cardiovascular complications due to KD [4]. As treatment options for IVIG-resistant KD patients, there have been reports of the usefulness of additional IVIG, corticosteroids, ulinastatin [5], plasma exchange [6] and anti-TNF-α therapy [7].

Looking back on the history of medical treatments for KD, corticosteroids, which are anti-inflammatory agents, were widely used prior to the establishment of standard IVIG therapy. However, corticosteroid monotherapy was subsequently reported to cause progression of CALs in some KD patients [8], and its use was long contraindicated. However, a retrospective study by Kobayashi et al. found that corticosteroid treatment was highly effective in suppressing CAL formation in KD [9]. That was especially true when the target patients were limited to severe KD, defined by a risk score predicting IVIG unresponsiveness [10]. Subsequent to that retrospective investigation [9], a randomized controlled study (RAISE study) demonstrated that initial combination therapy consisting of IVIG plus a corticosteroid for severe patients significantly reduced both the frequency of CAL formation and IVIG refractoriness [11]. That series of clinical studies led to reappraisal of the usefulness of initial combination therapy of IVIG plus a corticosteroid for patients with severe KD. However, the molecular mechanisms underlying the clinical benefits of that combination therapy remain unclear.

Several previous studies demonstrated an association between high-mobility group box-1 (HMGB1) and KD [12–14]. Serum levels of HMGB1 in KD patients were highest in the early acute phase before IVIG treatment and gradually decreased after fever reduction [12]. In addition, serum HMGB1 levels were significantly higher in IVIG-resistant KD than in IVIG-responsive KD [13]. That suggested that an elevated value of serum HMGB1 before IVIG treatment may be a useful biomarker for predicting IVIG-resistance in KD patients. HMGB1, a typical damage-associated molecular pattern (DAMP), is passively released by cells when they die or are damaged. These results thus suggest not only that HMGB1 may play an essential role in the pathogenesis of KD, but also that HMGB1 detected in the blood of KD patients may be derived from coronary arteries damaged due to acute inflammation. Therefore, more severe KD patients may have higher blood HMGB1 levels due to higher coronary artery damage levels.

In the course of endothelial damage, IL-1α, another DAMP, is also thought to be released from damaged cells. Our earlier study aimed at elucidating the mechanism of IVIG’s anti-inflammatory effects on coronary artery inflammation [15] found that high-dose IgG treatment specifically suppressed TNF-α-induced expression of IL-6 and granulocyte-colony stimulating factor (G-CSF), which are known to be crucially associated with the pathogenesis of acute KD [16, 17], in human coronary artery endothelial cells (HCAECs). However, that earlier study found that high-dose IgG treatment showed almost no effect on IL-6 and G-CSF expression when HCAECs were stimulated with IL-1β [15]. Therefore, we made the following two hypotheses. First, IL-1α released due to coronary artery damage plays critical roles in IVIG resistance in severe KD patients. Second, early introduction of a corticosteroid to standard IVIG therapy may be beneficial through suppression of coronary artery damage and damage-induced IL-1α release. To test these hypotheses, in the present study, we first examined high-dose IgG and dexamethasone (DEX), a corticosteroid, for differences in their effects on HCAECs’ release of DAMPs, including HMGB1 and IL-1α; we then compared their anti-inflammatory effects on HCAECs. Our present findings may help explain the mechanisms by which initial combination therapy using IVIG and a corticosteroid is effective in preventing IVIG resistance in KD patients.

## Methods

### Reagents

Recombinant human TNF-α, IL-1α and IL-1β were purchased from PeproTech (Rocky Hill, NJ, USA). DEX was purchased from Sigma-Aldrich (St. Louis, MO, USA). A human immunoglobulin preparation (Venoglobulin IH; USA) was provided by Japan Blood Products Organization (JB: Tokyo, Japan).

### Cell culture and treatment

HCAECs were purchased from Lonza (Walkersville, MD, USA) and maintained exactly as recommended by the manufacturer by using an EGM-2MV BulletKit (Lonza) at 37 °C in a humidified 5% CO2 atmosphere. We purchased two different HCAEC lots from individual donors (Lot Nos. 0000662152 and 0000626782) for this study, and all results were reproducible between these two lots. All the experiments described in this study were performed using second-passage cells.

Because the EGM-2MV BulletKit contains hydrocortisone, a corticosteroid, all the experiments described in this study were performed after hydrocortisone deprivation for at least 3 h to fairly evaluate the effects of DEX. More specifically, HCAECs were suspended in complete EGM-2MV medium and seeded into 96-well Optical-Bottom plates (Thermo Fisher Scientific; Waltham, MA, USA) at 1 × 10^4^ cells/well for apoptosis assay, 48-well culture plates (IWAKI AGC Techno Glass; Shizuoka, Japan) at 2 × 10^4^ cells/well for quantitative PCR (qPCR) and ELISA, and 24-well culture plates (IWAKI AGC Techno Glass) at 5 × 10^4^ cells/well for Western blotting (WB). The cells were grown to 90% confluency. Prior to stimulation, the medium in each well was replaced with hydrocortisone-deprived EGM-2MV for at least 3 h. The medium in each well was then replaced with hydrocortisone-deprived EGM-2MV medium containing the stimulant(s) and/or pharmacological agent(s) such as DEX and IVIG, as indicated in the figure legends.

### Elisa

The concentrations of HMGB1, IL-1α, IL-6 and G-CSF proteins in cell-free supernatants were measured with specific ELISA kits (R&D Systems; Minneapolis, MN, USA) in accordance with the manufacturer’s instructions.

### qPCR

Total RNA extraction from HCAECs, cDNA synthesis and qPCR were performed as previously described [18]. Primer sets for five genes were synthesized at Fasmac (Kanagawa, Japan): HMGB1 (sense, 5′-AGA AGT GCT CAG AGA GGT GGA-3′; antisense, 5′-CCT TTG GGA GGG ATA TAG GTT-3′), IL-1α (sense, 5′-CAA CCA GTG CTG AAG GAG-3′; antisense, 5′-TGC CGT GAG TTT CCC AGA AG-3), IL-6 (sense, 5′-CAA TAA CCA CCC CTG ACC CA-3′; antisense, 5′-GCG CAG AAT GAG ATG AGT TGT C-3′), G-CSF (sense, 5′-TGC TTA GAG CAA GTG AGG AAG ATC-3′; antisense, 5′-GCA CAC TCA CTC ACC AGC TTC T-3′), and β-actin (sense, 5′-CCC AGC CAT GTA CGT TGC TAT-3′; antisense, 5′-TCA CCG GAG TCC ATC ACG AT-3′). To determine the exact copy numbers of the five target genes, quantified concentrations of the purified PCR products of HMGB1, IL-1α, IL-6, G-CSF and β-actin were serially diluted and used as standards in each experiment. We used an aliquot of cDNA equivalent to 2 ng of each total RNA sample for each qPCR. The expression levels of mRNA were normalized to the β-actin level in each sample.

### WB

Whole cells were extracted with 200 μl of NuPAGE LDS sample buffer (Invitrogen; Carlsbad, CA, USA) containing 5% 2-mercaptethanol and then lysed by sonication. Equal amounts of whole-cell lysates were separated by SDS-PAGE (5–15% Ready Gels, Bio-Rad; Hercules, CA, USA) and transferred to PVDF membranes (Trans-Blot® Turbo™ Transfer System; Bio-Rad). WB was performed using the following antibodies (Abs) in accordance with the manufacturers’ instructions: biotinylated polyclonal goat anti-human IL-1α Ab (BAF200; R&D Systems) and polyclonal rabbit anti-heat shock protein 90 Ab (HSP90; Cell Signaling Technology; Danvers, MA, USA). ExtrAvidin-Peroxidase conjugate (Sigma-Aldrich) was used for detection of IL-1α protein, whereas peroxidase-linked anti-rabbit IgG Ab (Cell Signaling Technology) was used for detection of HSP90 protein.

### Caspase 3/7 activity assay

The caspase 3/7 activities in HCAECs were evaluated using the Caspase-Glo® 3/7 Assay System (Promega; Madison, WI, USA) in accordance with the manufacturer’s instructions. The data were collected using a multimode microplate reader (ARVO™, Perkin Elmer; Waltham, MA, USA).

### Statistical analysis

All data are presented as the mean ± SD of triplicate samples. Differences between groups were analyzed using ANOVA with Bonferroni’s post hoc test and were considered to be significant when *P* < 0.05.

## Results

### Effects of high-dose IgG and DEX on cellular damage to, and HMGB1 protein release by, HCAECs stimulated with inflammatory cytokines

**Fig. 1:**
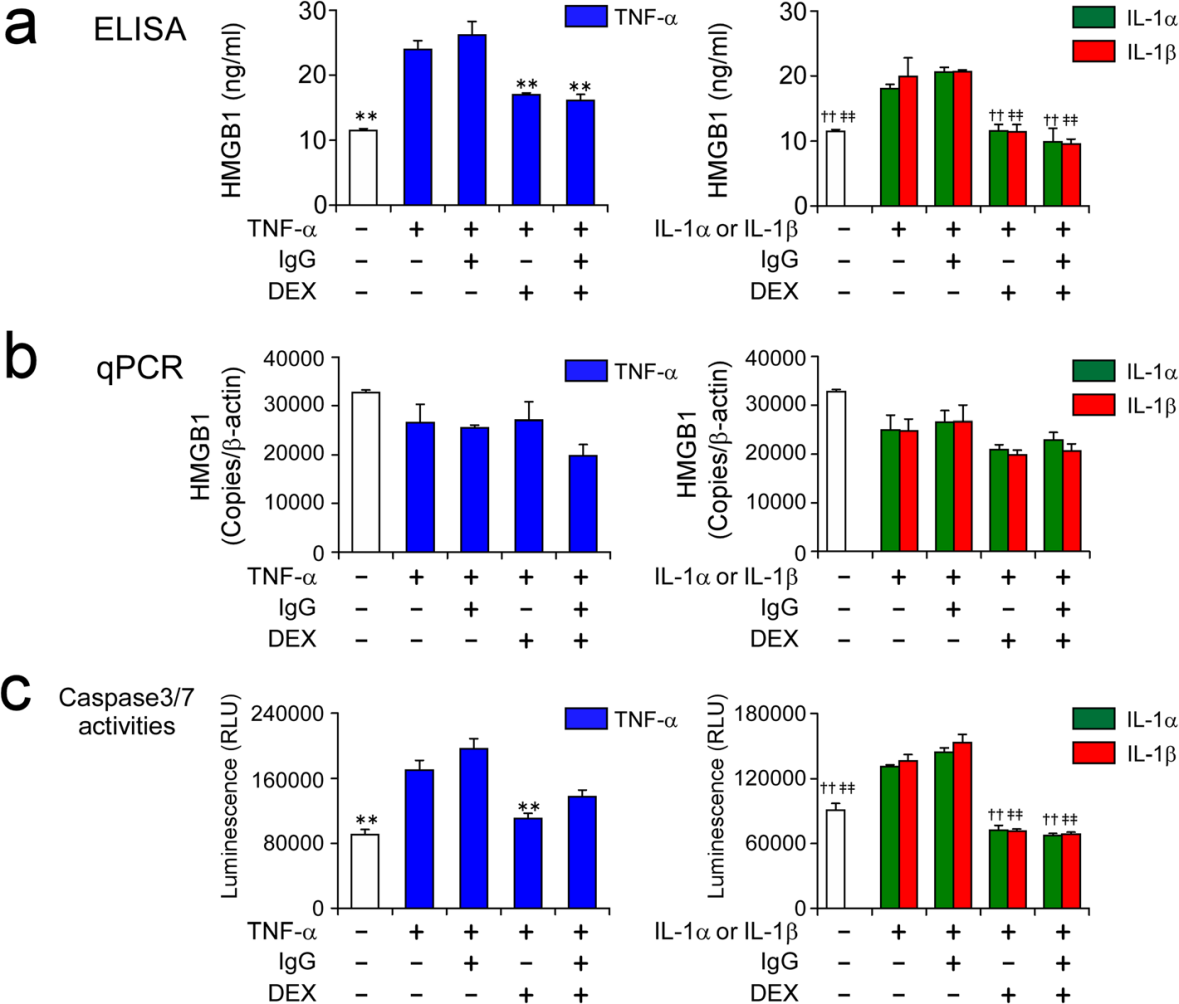
DEX, but not high-dose IgG, inhibits cellular damage to, and HMGB1 protein release by, HCAECs in response to inflammatory stimuli. HCAECs were stimulated with 100 ng/ml of TNF-α, or 10 ng/ml of IL-1α or IL-1β, for 24 h in the presence and absence of 10 mg/ml IgG and 1000 nM DEX, alone or in combination. Protein concentrations of HMGB1 in the culture supernatants (**a**), and HMGB1 mRNA levels (**b**) and caspase 3/7 activities in HCAECs (**c**) were measured by ELISA, qPCR and the Caspase-Glo 3/7 Assay System, respectively. Data are shown as the mean ± SD of triplicate samples and are representative of two individual experiments using HCAEC lots from different donors. **P < 0.01 compared with 100 ng/ml TNF-α; ††*P* < 0.01 compared with 10 ng/ml IL-1α; and ‡‡*P* < 0.01 compared with 10 ng/ml IL-1β

HCAECs were stimulated with 100 ng/ml of TNF-α, or 10 ng/ml of IL-1α or IL-1β for 24 h in the presence and absence of 10 mg/ml IgG and 1000 nM DEX, alone or in combination. The concentrations of cytokines and DEX were determined based on the results of preliminary experiments (Additional files 1 and 2: Figs. S1 and S2). Damage to the HCAECs due to the inflammatory stimuli was evaluated by their release of HMGB1 protein. It is of note that HMGB1 protein release by the HCAECs was not suppressed at all by IgG treatment, whereas it was significantly suppressed by DEX treatment, for each of the tested cytokine stimuli (Fig. 1a). The HMGB1 mRNA expression level in the HCAECs showed no significant changes under any conditions (Fig. 1b). Furthermore, the cytokine-enhanced caspase 3/7 activities in the HCAECs were inhibited by DEX treatment, but not by IgG treatment (Fig. 1c).

### Effects of high-dose IgG and DEX on IL-1α expression in HCAECs stimulated with inflammatory cytokines

**Fig. 2:**
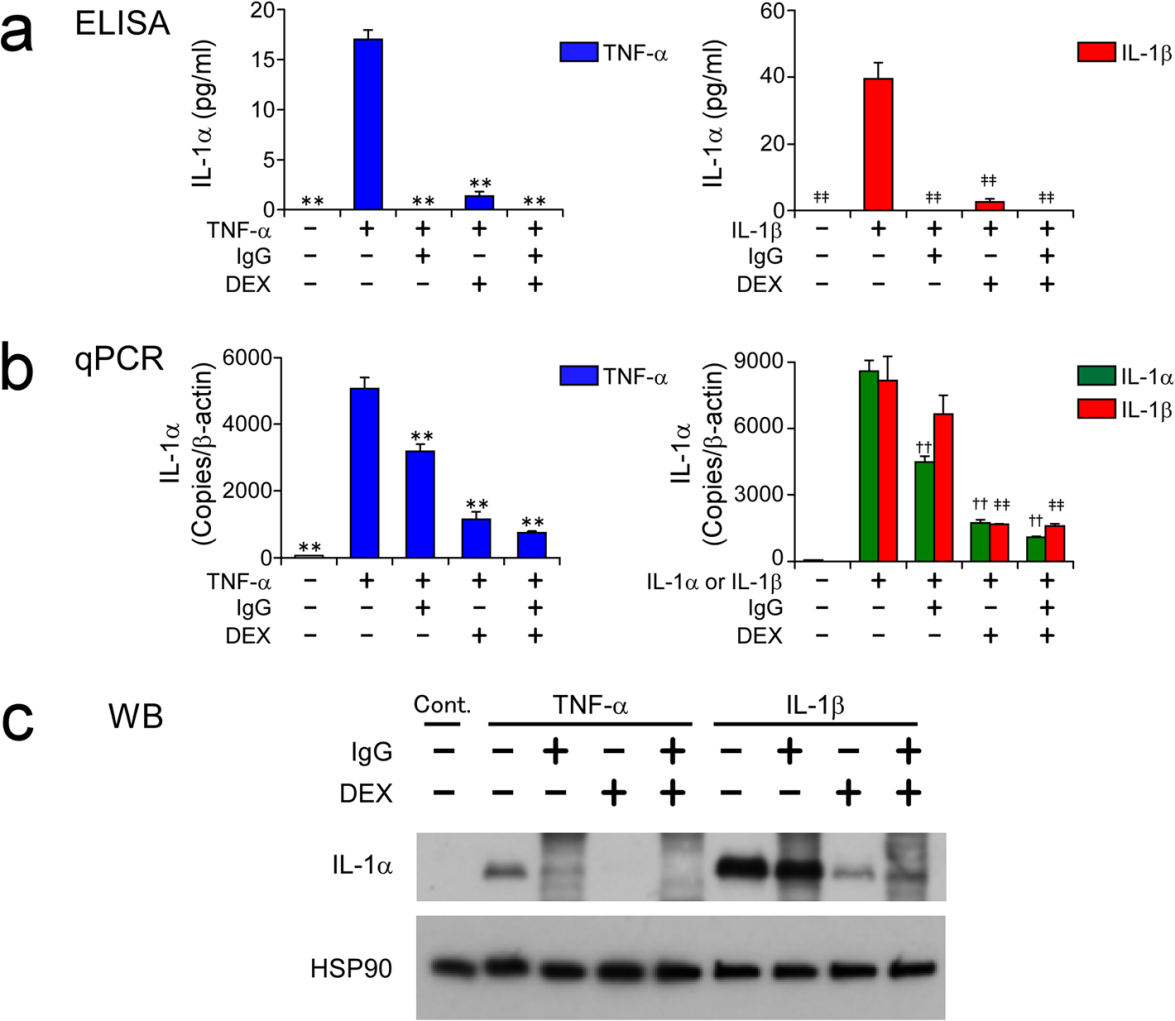
DEX inhibits expression and release of IL-1α by HCAECs in response to inflammatory stimuli. HCAECs were stimulated with 100 ng/ml of TNF-α, or 10 ng/ml of IL-1α or IL-1β, for 48 h in the presence and absence of 10 mg/ml IgG and 1000 nM DEX, alone or in combination. Protein concentrations of IL-1α in HCAEC culture supernatants (**a**) and mRNA levels of IL-1α in HCAECs (**b**) were measured by ELISA and qPCR, respectively. Whole-cell lysates of HCAECs were subjected to WB analysis of the expression of IL-1α and heat shock protein 90 (HSP90; as a loading control) (**c**). Data shown in **a** and **b** are the mean ± SD of triplicate samples. All data are representative of two individual experiments using HCAEC lots from different donors. **P < 0.01 compared with 100 ng/ml TNF-α; ††*P* < 0.01 compared with 10 ng/ml IL-1α; and ‡‡*P* < 0.01 compared with 10 ng/ml IL-1β

Both IL-1α and IL-1β are cytokines belonging to the IL-1 family, bind to the same receptors and induce the same biological functions, but their production mechanisms by cells are different [19]. Briefly, secretion of activated IL-1β requires Nod-like receptor (NLR) family, pyrin domain-containing 3 (NALP3)/caspase 1-dependent inflammasome activation [20], whereas IL-1α is a DAMP, like HMGB1 [19]. We next examined the effects of IgG and DEX on cytokine-induced release of IL-1α. Both TNF-α and IL-1β stimulation induced significant release of IL-1α into the HCAEC culture supernatants, but DEX treatment effectively inhibited that release (Fig. 2a). DEX treatment also significantly inhibited both mRNA (Fig. 2b) and intracellular protein expression of IL-1α (Fig. 2c). Curiously, IL-1α protein could hardly be detected in the IgG-treated HCAEC culture supernatants (Fig. 2a), but IgG treatment was less effective than DEX in inhibiting both mRNA (Fig. 2b) and intracellular protein expression (Fig. 2c) of IL-1α induced by inflammatory stimuli. We posited that high-dose IgG might interfere with detection of IL-1α protein in this ELISA system. To confirm that, we added recombinant IL-1α protein equivalent to the maximum IL-1α level released by HCAECs in this study (approximately 100 pg/ml; see Additional file 1: Fig. S1, middle right graph) to culture medium samples, to which IgG was then added at concentrations of 0 to 10 mg/ml. As posited, ELISA examination of those samples found that the detectability of IL-1α protein decreased with increasing IgG concentration, and it could not be detected at all at 10 mg/ml of IgG (Additional files 3: Figs. S3). These findings are in agreement with an earlier finding that anti-IL-1α antibody is contained in human immunoglobulin preparations [21].

### Effects of high-dose IgG and DEX on expression of IL-6 and G-CSF in HCAECs stimulated with inflammatory cytokines

**Fig. 3:**
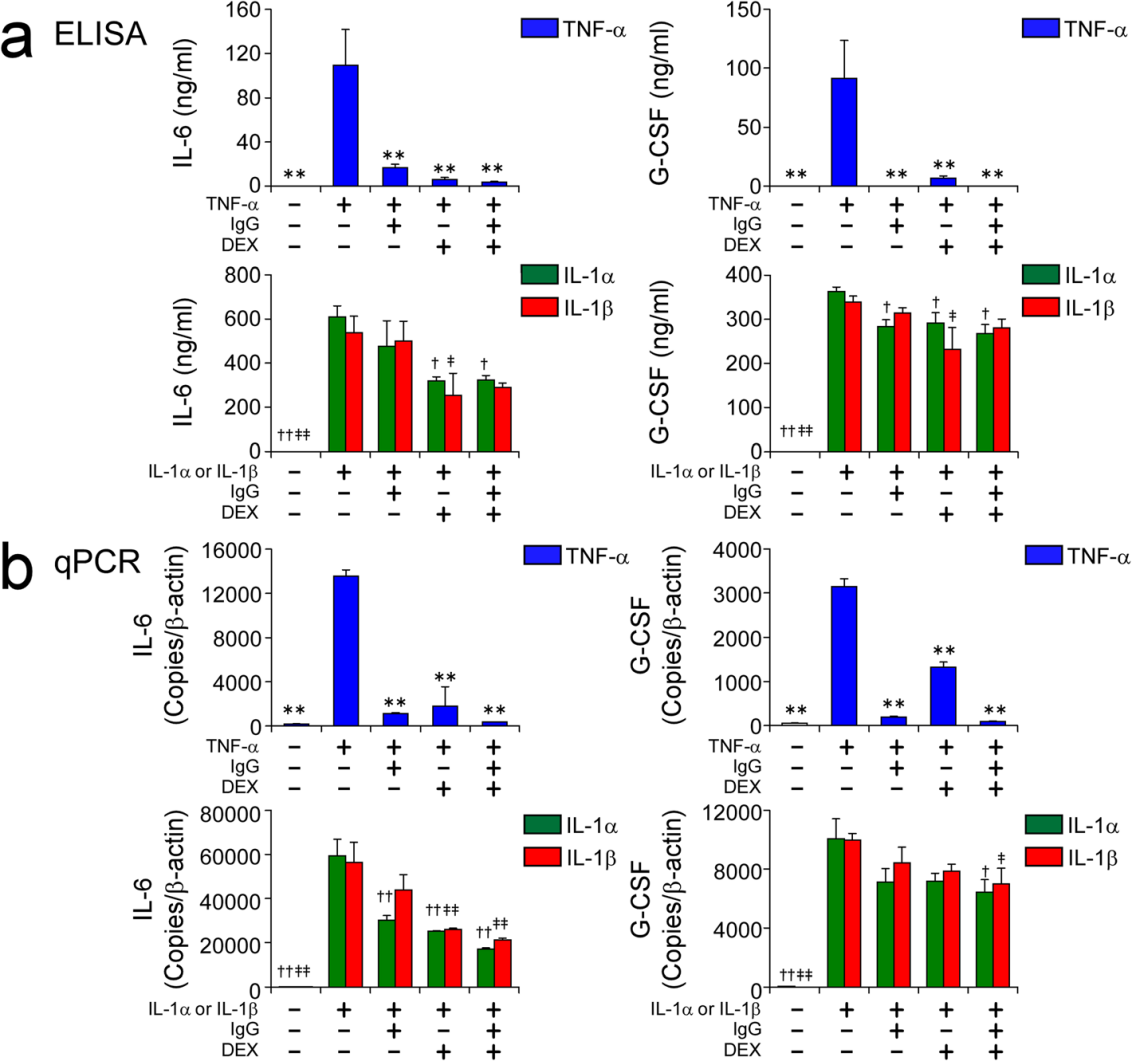
Effects of DEX and high-dose IgG on inflammatory cytokine-induced expression of IL-6 and G-CSF in HCAECs. HCAECs were stimulated with 100 ng/ml of TNF-α, or 10 ng/ml of IL-1α or IL-1β, for 48 h in the presence and absence of 10 mg/ml IgG and 1000 nM DEX, alone or in combination. Protein concentrations of IL-6 and G-CSF in the culture supernatants (**a**) and mRNA levels of IL-6 and G-CSF (**b**) in HCAECs were measured by ELISA and qPCR, respectively. Data are shown as the mean ± SD of triplicate samples and are representative of two individual experiments using HCAEC lots from two different donors. **P < 0.01 compared with 100 ng/ml TNF-α; †*P* < 0.05 and ††*P* < 0.01 compared with 10 ng/ml IL-1α; and ‡*P* < 0.05 and ‡‡*P* < 0.01 compared with 10 ng/ml IL-1β

We next examined the effects of high-dose IgG and DEX on cytokine-induced expression of IL-6 and G-CSF, which are crucially involved in the pathogenesis of KD [16, 17]. We previously demonstrated that both IL-6 and G-CSF mRNA expressions were decreased the most by IVIG treatment among all TNF-α-inducible genes in HCAECs [15]. Consistent with that report, both IgG and DEX effectively inhibited TNF-α-induced production of IL-6 and G-CSF by HCAECs (Fig. 3a, upper graphs). On the other hand, similar to our previous finding that IL-1β stimulation led to IVIG-resistant production of IL-6 and G-CSF by HCAECs [15], we newly found that IL-1α induced exactly the same IVIG-resistant production of those cytokines (Fig. 3a, lower graphs). DEX treatment partially inhibited IL-1 s-induced IL-6 production by HCAECs (Fig. 3a, lower left graph), but its effect was much weaker than that of TNF-α (Fig. 3a, upper left graph). In addition, G-CSF production induced by IL-1 s was hardly inhibited by DEX treatment, similar to the case of IVIG treatment (Fig. 3a, lower right graph). The mRNA expression patterns for both IL-6 and G-CSF (Fig. 3b) were in line with their protein production patterns (Fig. 3a).

### Inhibitory kinetics of high-dose IgG and DEX on the cytokine-induced production/release of IL-6, G-CSF and IL-1α by HCAECs

**Fig. 4:**
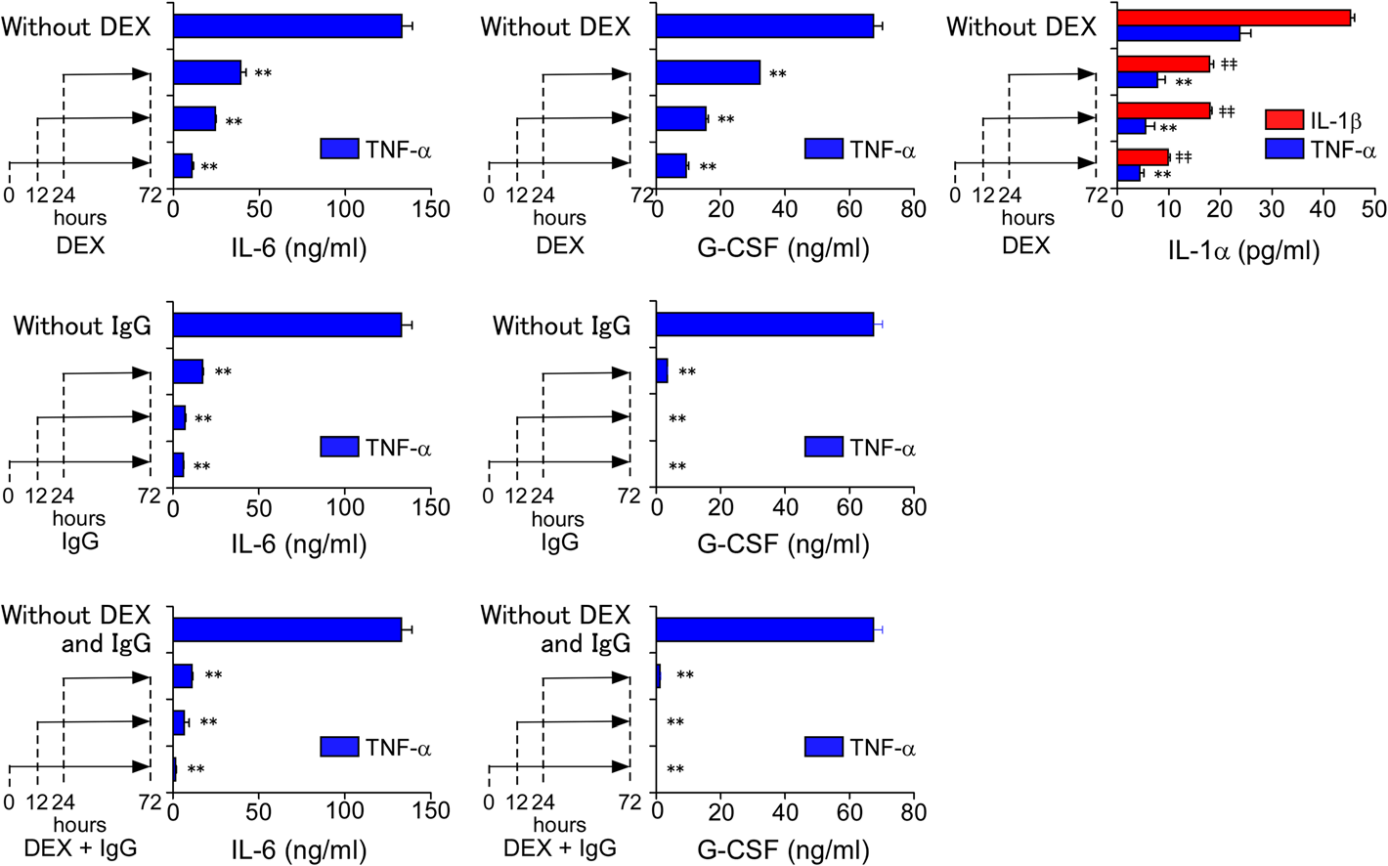
Inhibitory kinetics of DEX and high-dose IgG on cytokine-induced production/release of IL-6, G-CSF and IL-1α by HCAECs. HCAECs were stimulated with 100 ng/ml of TNF-α or 10 ng/ml of IL-1β alone (without drugs) for 72 h and then treated with 1000 nM of DEX and/or 10 mg/ml of IgG at 0, 12 and 24 h after stimulation. The cell supernatants were collected at 72 h after cytokine stimulation. The protein concentrations of IL-6, G-CSF and IL-1α in the culture supernatants were measured by ELISA. Data are shown as the mean ± SD of triplicate samples and are representative of two individual experiments using HCAEC lots from two different donors. **P < 0.01 compared with 100 ng/ml TNF-α; and ‡‡*P* < 0.01 compared with 10 ng/ml IL-1β

We next investigated the inhibitory effects of high-dose IgG and DEX at various time points after cytokine stimulation. HCAECs were stimulated with TNF-α or IL-1β alone, followed by treatment with DEX and/or IgG at 0, 12 and 24 h later (Fig. 4). The cell supernatants were collected at 72 h after the cytokine stimulation. The effects of DEX and IgG on TNF-α-induced production of IL-6 and G-CSF (anti-inflammatory effects) were strongest when the drugs were administered at the start of stimulation (0 h). In addition, the combination of DEX and IgG showed the strongest anti-inflammatory effects, even though they were added later (Fig. 4, lower graphs vs. middle and upper graphs). DEX alone also effectively inhibited IL-1α release induced by TNF-α or IL-1β stimulation, but its effects were stronger when it was added earlier (Fig. 4, upper right graph).

## Discussion

In this study, we used HCAECs, as a mimic of KD, to study the possible mechanisms responsible for the clinical benefits of adding a corticosteroid to standard IVIG therapy for patients with severe KD. We first examined the effects of high-dose IgG and DEX, a synthetic corticosteroid, on cellular damage to HCAECs caused by inflammatory stimuli. The degree of cellular damage was evaluated by the level of HMGB1 protein released by HCAECs in response to stimulation with three inflammatory cytokines, TNF-α, IL-1α and IL-1β. We found that DEX, but not IgG, significantly inhibited the release of HMGB1 by HCAECs (Fig. 1a). Furthermore, there was no significant change in HMGB1 mRNA expression levels in HCAECs under any of the test conditions (Fig. 1b). This suggested that the elevated HMGB1 protein in the culture supernatants was not newly-synthesized protein, but passively-released protein due to cellular damage caused by the inflammatory cytokine stimulation. In fact, the TNF-α-induced HMGB1 release from HCAECs was not inhibited by treatment with 1 μM monensin A (Golgi-Stop reagent; data not shown). Consistent with previous reports using human umbilical vein endothelial cells (HUVECs) [22, 23], DEX effectively inhibited endothelial cell apoptosis by reducing caspase 3/7 activities in HCAECs (Fig. 1c).

Interestingly, a Korean research group recently reported that *HMGB1* single nucleotide polymorphisms (SNPs) were significantly associated with both IVIG resistance and CAL formation in Korean KD patients, but not with KD susceptibility [14]. Those findings suggest that the amount of HMGB1 released from damaged endothelial cells might be related to the severity and complications in KD patients, but not their susceptibility to KD. As far as we examined, HMGB1 failed to directly induce an inflammatory response by HCAECs (data not shown). However, once released into the extracellular milieu, HMGB1 reportedly activated monocytes/macrophages to produce multiple proinflammatory cytokines [24, 25] and exerted several inhibitory effects on regulatory T cell activities [26, 27]. Thus, extracellular HMGB1 might act on various types of leukocytes, perhaps leading to KD aggravation. Blood HMGB1 levels may reflect the degree of coronary vascular endothelial cell damage in KD patients. Accordingly, stratifying patients by adding the blood HMGB1 level to the existing risk score(s) for predicting IVIG resistance may increase the probability of success of combination therapy consisting of IVIG plus a corticosteroid.

Unlike HMGB1, IL-1α—another DAMP—was significantly induced in HCAECs at the mRNA expression level by inflammatory stimuli (Fig. 2b vs. Figure 1b). Furthermore, consistent with the results of qPCR, DEX effectively inhibited cytokine-induced intracellular IL-1α protein (Fig. 2c). Similar to HMGB1, TNF-α-induced IL-1α release by HCAECs was not inhibited by treatment with 1 μM monensin A (Golgi-Stop reagent; data not shown), indicating that intracellularly accumulated IL-1α protein was passively released from damaged HCAECs. Therefore, we speculate that the decrease in IL-1α protein seen with DEX (Fig. 2a) was due to the combination of DEX’s suppression of IL-1α mRNA expression (Fig. 2b) and its anti-cytotoxic effect on HCAECs (Fig. 1). Notably, IL-1α can induce a strong inflammatory response (IL-6 and G-CSF production) comparable to that seen with IL-1β, even at lower concentrations compared to TNF-α (Additional file 1: Fig. S1). In order to compare and evaluate the efficacy of corticosteroid and IgG under conditions with similar levels of inflammation and cytotoxicity, 100 ng/ml of TNF-α and 10 ng/ml of IL-1 s were used as inflammatory stimuli in this study.

Although we previously reported that IVIG treatment hardly inhibited IL-1β-induced IL-6 and G-CSF production [15], it should be noted that IL-1α stimulation resulted in IVIG resistance (Fig. 3a, lower graphs). Several recent studies reported an association between IL-1 s and IVIG resistance in KD patients. In a microarray study using whole-blood RNA, IL-1-associated signaling pathways were upregulated in IVIG-resistant KD patients compared to IVIG-responsive patients [28]. Two previous case reports suggested a beneficial effect of anakinra (an IL-1R antagonist that blocks the activity of both IL-1α and IL-1β) on IVIG-resistant KD [29, 30]. Based on those findings, clinical trials of IL-1 blockade for IVIG-resistant KD patients are being conducted in Western Europe and the USA [31].

Like IL-1 s, TNF-α has been reported to be involved in the pathogenesis of KD. Serum levels of TNF-α were significantly elevated and correlated with the incidence of CALs in acute KD patients [32, 33]. Furthermore, TNF-α blockade effectively prevented the development of coronary vasculitis in murine models of KD [34, 35]. In fact, a clinical trial of an anti-TNF monoclonal antibody (mAb) showed clinical effectiveness, including reduced fever duration and CAL formation [36]. Thus, although both TNF-α and IL-1 s have been clearly implicated as key pathogenic cytokines in KD, there is currently limited understanding of whether their roles are distinct or overlapping. Stock et al. recently addressed this issue in a murine model of KD and provided evidence that TNF-α and IL-1 s play temporally distinct and non-redundant roles in driving cardiac inflammation [37]. Specifically, TNF-α, but not IL-1 s, was essential for the development of acute-phase myocarditis, whereas IL-1 s were indispensable for the subsequent development of coronary vasculitis [37]. These findings suggest the possibility that TNF-α is more critical for the onset of KD, whereas IL-1 s may be more crucially involved in the progression and prognosis of KD than TNF-α. Taken together with our present findings, administration of a corticosteroid as early as possible may contribute to suppression of KD progression by inhibiting the expression and/or release of IL-1 s.

Corticosteroids are widely used as potent anti-inflammatory drugs to treat various inflammatory diseases. As a preliminary experiment, we examined for concentration-dependency of DEX’s inhibitory effects on IL-6 and G-CSF production and IL-1α release induced by inflammatory stimuli (Additional file 2: Fig. S2). We found that the inhibitory effects of DEX were indeed concentration-dependent, but they almost reached a plateau at 100 nM to 10,000 nM of DEX. When the blood concentration of corticosteroid used in the RAISE study [11] is converted to DEX on the basis of the titer, it is about 10,000 nM. However, sufficient effects were observed even at 100 nM and 1000 nM DEX in our in vitro experiment (Additional file 2: Fig. S2), and for that reason we used 1000 nM DEX in this study.

Corticosteroids are known to suppress nuclear factor kappa B (NF-κB), which promotes transcriptional activation of various inflammatory genes, including IL6 [38–40]. We previously demonstrated that IVIG did not inhibit activation of NF-κB, whereas it significantly inhibited activation of another transcription factor, CCAAT/enhancer-binding protein delta (C/EBPδ) [15], as well as C/EBPβ [41]. Therefore, synergistic effects between a corticosteroid and IgG seem likely because their anti-inflammatory mechanisms apparently involve non-overlapping pathways. Indeed, these drugs were more effective in suppressing IL-6 production and IL-1α release when added immediately after the inflammatory stimulation (Fig. 4). Thus, adding a corticosteroid to standard IVIG therapy at an early stage of inflammation in KD patients may have a better anti-inflammatory effect by inhibiting both KD-related cytokine production and release of IVIG-refractory factors, including HMGB1 and IL-1α.

## Conclusions

**Fig. 5:**
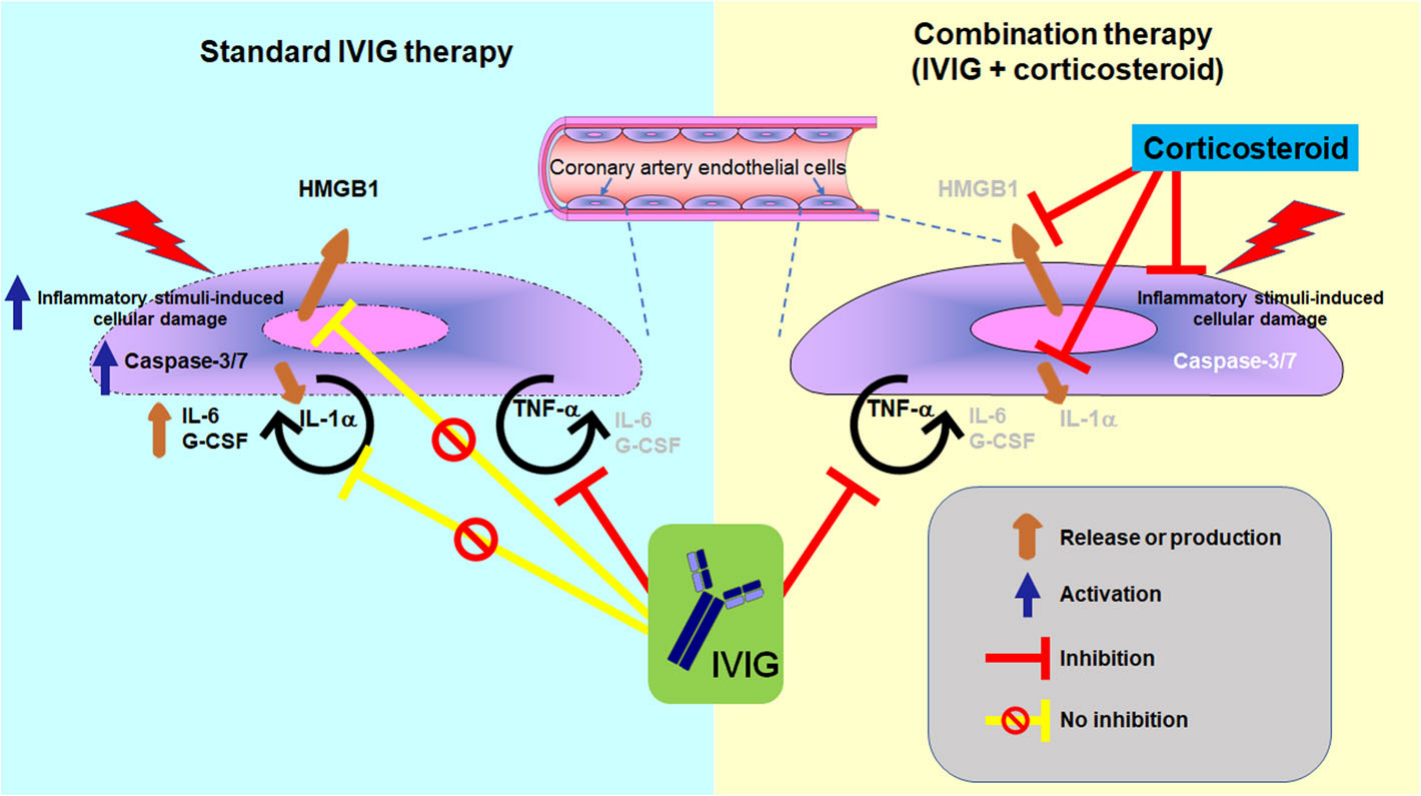
Schematic illustration of the functional benefits of combination therapy consisting of standard IVIG plus a corticosteroid for severe KD patients

To summarize our findings, a schematic illustration is presented in Fig. 5. We sought to elucidate the benefits of combination therapy consisting of a corticosteroid and IVIG from a mechanistic perspective, especially as an initial treatment for KD patients who are predicted to be severely resistant to IVIG. Our findings indicate the possibility of benefits arising from that combination therapy. Most important, a corticosteroid, but not IgG, can potentially prevent inflammatory stimulus-induced coronary artery endothelial cell damage. That would contribute to inhibiting the inflammatory stimulus-induced release of DAMPs, including HMGB1 and IL-1α, which may be involved in IVIG resistance in KD patients. Furthermore, the corticosteroidsignificantly inhibited expression of IL-1α (Fig. 2) as well as IL-1β [15]. Both cytokines can induce IVIG-resistant production of IL-6 and G-CSF by HCAECs (Fig. 3a), probably contributing to IVIG resistance in KD patients [28–31]. Since such effects of a corticosteroid would probably help prevent the progression of IVIG resistance in KD, it would be better to start the combination therapy as soon as possible, especially for KD patients who are predicted to be IVIG-resistant. KD is a systemic inflammatory disease, and leukocytes also play a crucial role in its pathogenesis. In this study, however, we used HCAECs as an in vitro mimic of KD. Therefore, the involvement of leukocytes cannot be considered in this model, which is a limitation of this study. Nevertheless, our present findings may, from the mechanistic viewpoint, at least partly explain the clinical effectiveness of combination therapy consisting of IVIG plus a corticosteroid for severe KD patients.

## Supplementary information

**Additional file 1: Figure S1.** Increase in concentrations of IL-6, G-CSF and IL-1α proteins depending on the concentration and duration of inflammatory stimulation of HCAECs.

**Additional file 2: Figure S2.** Concentration dependence of inhibitory effects of DEX on the inflammatory cytokine-induced production of IL-6, G-CSF and IL-1α by HCAECs.

**Additional file 3: Figure S3.** High-dose IgG interfered with detection of IL-1α protein by ELISA.

## Data Availability

All data generated or analyzed during this study are included in this published article and its supplementary information files.

## Abbreviations

CALs: Coronary artery lesions
C/EBP: CCAAT/enhancer-binding protein
DAMPs: Damage-associated molecular patterns
DEX: Dexamethasone
G-CSF: Granulocyte-colony stimulating factor
HCAECs: Human coronary artery endothelial cells
HMGB1: High-mobility group box-1
HSP90: Heat shock protein 90
IVIG: Intravenous immunoglobulin
KD: Kawasaki disease
NF-κB: Nuclear factor kappa B
qPCR: Quantitative PCR
WB: Western blotting
