# Supplementary material for: Functional benefits of corticosteroid and IVIG combination therapy in a coronary artery endothelial cell model of Kawasaki disease
Source: Pediatr Rheumatol Online J. 2020 Oct 6;18:76. doi: 10.1186/s12969-020-00461-6 (PMC7539408; doi:10.1186/s12969-020-00461-6)
Supplement: Supplementary file 1 — Additional file 1: Figure S1. Increase in concentrations of IL-6, G-CSF and IL-1α proteins depending on the concentration and duration of inflammatory stimulation of HCAECs. [file 12969_2020_461_MOESM1_ESM.docx]

**Additional file 1**


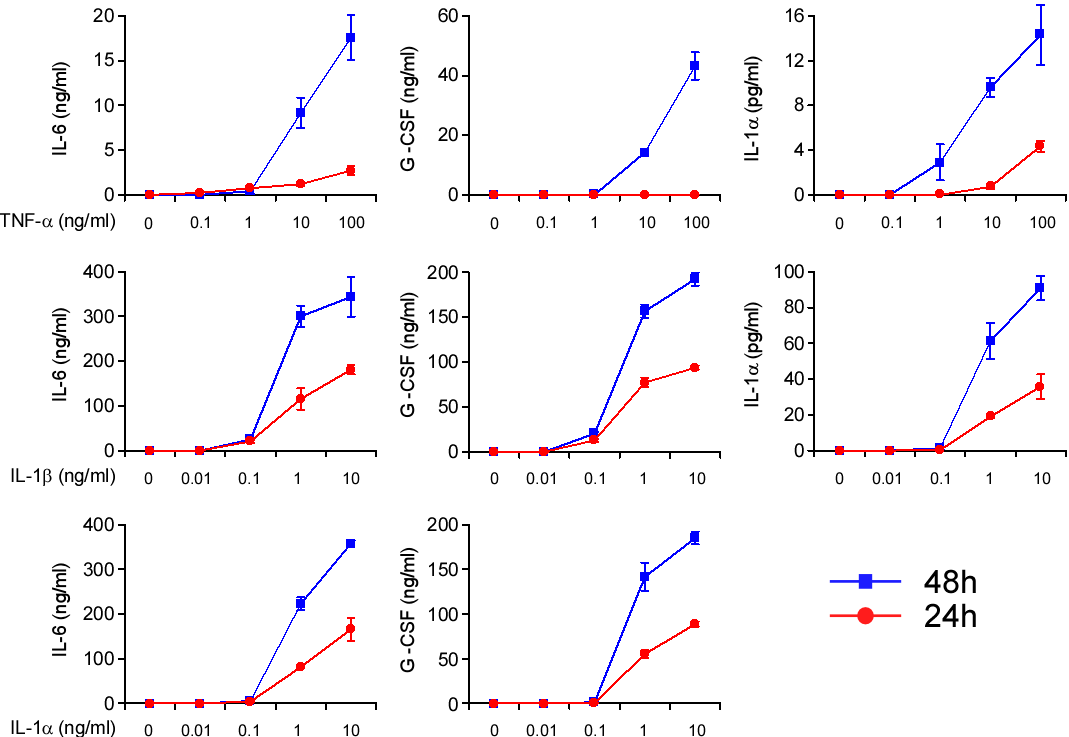


**Fig. S1: Increase in concentrations of IL-6, G-CSF and IL-1α proteins depending on the concentration and duration of inflammatory stimulation of HCAECs**

HCAECs were stimulated with 0.1, 1, 10, 100 ng/ml of TNF-α, or 0.01, 0.1, 1, 10 ng/ml of IL-1α or IL-1β for 24 or 48 h. Protein concentrations of IL-6, G-CSF and IL-1α in the culture supernatants were measured by ELISA. Data are shown as the mean ± SD of triplicate samples and are representative of two individual experiments using HCAEC lots from different donors.
