# Supplementary material for: Functional benefits of corticosteroid and IVIG combination therapy in a coronary artery endothelial cell model of Kawasaki disease
Source: Pediatr Rheumatol Online J. 2020 Oct 6;18:76. doi: 10.1186/s12969-020-00461-6 (PMC7539408; doi:10.1186/s12969-020-00461-6)
Supplement: Supplementary file 2 — Additional file 2: Figure S2. Concentration dependence of inhibitory effects of DEX on the inflammatory cytokine-induced production of IL-6, G-CSF and IL-1α by HCAECs. [file 12969_2020_461_MOESM2_ESM.docx]

**Additional file 2**

**
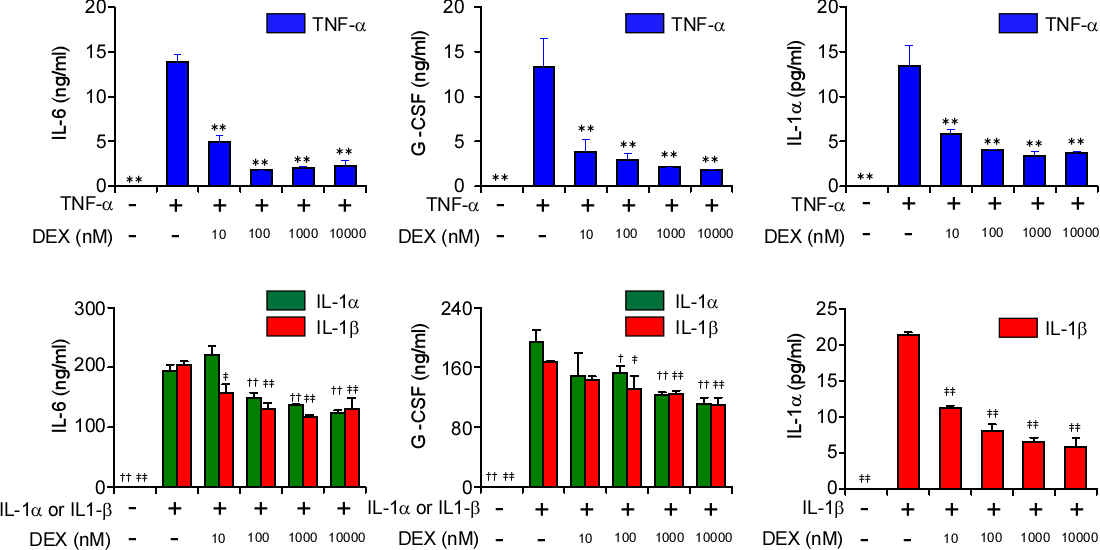
**

**Fig. S2: Concentration dependence of inhibitory effects of DEX on the inflammatory cytokine-induced production of IL-6, G-CSF and IL-1α by HCAECs**

HCAECs were stimulated with 100 ng/ml of TNF-α, or 10 ng/ml of IL-1α or IL-1β for 48 h in the presence and absence of 10, 100, 1000 and 10000 µM of DEX. Protein concentrations of IL-6, G-CSF and IL-1α in the culture supernatants were measured by ELISA. Data are shown as the mean ± SD of triplicate samples and are representative of two individual experiments using HCAEC lots from different donors. ***P* <0.01 compared with 100 ng/ml TNF-α; †*P* <0.05 and ††*P* <0.01 compared with 10 ng/ml IL-1α; and ‡*P* < 0.05 and ‡‡*P* < 0.01 compared with 10 ng/ml IL-1β.
