# Supplementary material for: Functional benefits of corticosteroid and IVIG combination therapy in a coronary artery endothelial cell model of Kawasaki disease
Source: Pediatr Rheumatol Online J. 2020 Oct 6;18:76. doi: 10.1186/s12969-020-00461-6 (PMC7539408; doi:10.1186/s12969-020-00461-6)
Supplement: Supplementary file 3 — Additional file 3: Figure S3. High-dose IgG interfered with detection of IL-1α protein by ELISA. [file 12969_2020_461_MOESM3_ESM.docx]

**Additional file 3**

**
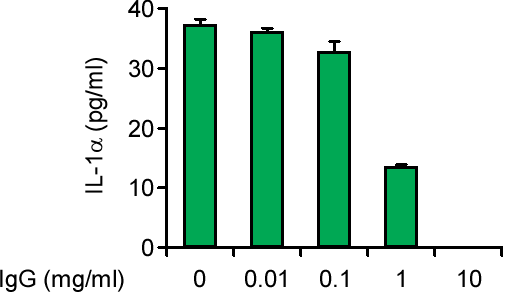
**

**Fig. S3: High-dose IgG interfered with detection of IL-1α protein by ELISA**

One hundred pg/ml of recombinant IL-1α protein and IgG in the range of 0 to 10 mg/ml were added to the culture medium for HCAECs (EGM-2MV complete medium), followed by incubation at room temperature for 1 hour. The protein concentrations of IL-1α in the culture mixtures were then measured by ELISA. Data are shown as the mean ± SD of triplicate samples. Spearman’s rank correction coefficient (*Rs*) was applied to determine correlations between the concentrations of IgG and IL-1α protein, and *Rs* = -1.
